# Enhanced Recovery: A Decade of Experience and Future Prospects at the Mayo Clinic

**DOI:** 10.3390/healthcare9050549

**Published:** 2021-05-08

**Authors:** Jenna K. Lovely, David W. Larson

**Affiliations:** Mayo Clinic, Rochester, MN 55902, USA; larson.david2@mayo.edu

**Keywords:** enhanced recovery, surgical care, colorectal surgery, quality improvement

## Abstract

This work aims to describe the implementation and subsequent learnings from the first decade after the full implementation of enhanced recovery pathway for colorectal surgery at a single institution. This paper will describe the diffusion efforts and plans through the Define, Measure, Analyze, Improve, Control (DMAIC) process of ongoing quality improvement and through research efforts. The information applies to all readers that provide surgical care within their organization as the fundamental principles of enhanced recovery for surgery are applicable regardless of the setting.

## 1. Problem Description

Starting in 2008, it was recognized that while well-established literature supported principles of enhanced recovery, full implementation of those principles had yet to be delivered to our patients. Enhanced recovery is referred to under different names, known as Enhanced Recovery After Surgery (ERAS^®^), rapid recovery, or earlier referred to Fast-track programs [1,2,3]. Implementation has developed over 20 years after described by Kehlet in 1997 [4]. Several groups across the world have contributed to over 1000 PubMed search articles demonstrating benefits with Enhanced Recovery principles, while the ERAS^®^ Society has developed numerous published guidelines covering specialty and sub-specialty surgeries [5,6,7,8,9,10,11,12,13,14,15,16,17]. The principles of enhanced recovery, when fully implemented, have been demonstrated to reduce the length of hospital stay (LOS), morbidity, and convalescence, without an increase in readmission rates or complications [4,18,19,20]. Enhanced recovery pathways (ERP) can be considered a Quality Improvement (QI) intervention and are an inter-professional and multimodal approach to care [21,22,23,24,25,26]. ERP seeks to optimize patient care before, during, and after surgery to minimize the surgical stress response. The pathways are multimodal and combine preoperative education, minimally invasive surgery, regional anesthetic techniques, multimodal opioid-sparing pain management, early feeding, and ambulation [27,28,29,30,31,32,33,34]. To address this problem of the gap from knowledge in literature and conceptual agreement to actualization in the practice [35], we first set out to develop and implement an institutional pathway for enhanced recovery. As the results of that implementation were known, we worked to address new problems and answer new research questions while also spreading to other surgical specialties.

After over a decade of a fully implemented enhanced recovery pathway [36,37], our institution has embarked on several innovations and quality improvement initiatives to continue to evolve toward the next new standard of innovative care [38,39,40]. The age-old project management challenges continue to impede delivering optimal care. Critical elements of the implementation [35,41,42] dissemination, and sustainability [43,44] are areas for all interested parties to engage.

The specific aim of this paper strives to provide those working to implement and adopt enhanced recovery pathways, the principles intended to stretch collaborative thinking and execute high value patient care. 

## 2. Methods

### 2.1. Standards of Care

The first stage was to fully implement the institution developed enhanced recovery pathway. This pathway was based on evidence-based principles and where no specific literature, existed, then by consensus developed agreement with interdisciplinary team members. The lead surgeon, anesthesiologist, clinical pharmacist, and clinical nurse specialist designed with input for logistics from variety stakeholders. Using combined methodology of 5 Whys, Value Stream Mapping and Failure Mode Effect analysis, the entire process was set for clinical excellence and operationally LEAN. These standards were then implemented in stages. First with one surgeon in minimally invasive practice for two weeks, then with a second surgeon for timeframe of two months. Daily tracking of the process occurred by the clinical pharmacist with follow up within the day for other team members as needed. All pathway elements were tracked manually. Preliminary results for inpatient metrics and for 30 day outcomes to date were analyzed. The overwhelming improvements led to full adoption for two surgeons for all patients and procedures in 2010. The process and outcome measures continued to be tracked in a prospectively maintained database, with ongoing automation added where able within the electronic environment. After 3 months of overall data, all surgeons in the Division were invited to fully adopt and be supported with the tracking and monitoring plan for implementation in place. With adoption, it was recognized that a more formal implementation expectation was needed. We worked to publish our results and started new research studies to answer questions to the specific themes about risks and complications that those hesitant were claiming. The data for renal insufficiency, elderly patients and those with inflammatory bowel disease were internally reviewed and then further studied. No harm, only benefit was shown for the surgeons. This Design, Measure, Analyze, Improve, Control (DMAIC) process continued for each area of low compliance [45]. In 2011, the Division approved and committed to full implementation of enhanced recovery as a practice standard. The outcomes of that implementation are described and highlighted that diet and fluids were key to outcomes [36,38]. This is consistent with findings from others [46,47,48] The lessons learned demonstrated that standardized care is about discovering best practice, implementing it, publishing the results, and teaching it to others. These principles of care translate, for patients and providers, into less waste and unnecessary delays in the system, patient flow and improves patient experience [49]. 

Standardization of care leads to improved pattern recognition of complications versus non-standardized care. Enhanced recovery pathways bring the patient through highly choreographed preoperative care, intraoperative care, PACU, post-operative care, discharge, and post-discharge follow-up. This fact allows one to recognize deviations from this standard which typically represent complications resulting in earlier intervention and, ultimately better outcomes [38,40,50,51,52]. In the rectal cancer patient population specifically, we learned that the patterns recognized on days 2–3 may result in early diagnosis and treatment of complications [40,53,54].

Another example may be when the Pharmacist may recognize that a certain amount of opioid medication was a signal for reassessment—patients requiring more than the usual needed attention. This attention can afford a new diagnosis of a complication or a reassessment in partnership with the pharmacist for another multimodal option and work to taper the opioid effectively [37,38,55,56]. 

Our Enhanced Recovery Pathway (ERP) provides for all the standard orders required for high value patient care during the first 48 h of post-op care until standardized discharge criteria have been met. After 48 h, the focus shifts to reassessment and recognizing alterations in patient status which may represent complications, or logistical barriers to discharge. 

In the Colorectal surgery practice, providers can shortlist complications to focus on as in many other surgical specialties. Prevention, early recognition, and optimal management of complications are critical. This focused list of complications includes postoperative ileus, surgical site infection, bleeding, anastomotic leak, venous thrombosis, acute kidney injury, atrial fibrillation, or acute myocardial infarction. Focusing on these issues ensure prevention and recognition, and an early management treatment resulting in a smoother implementation of treatment. 

### 2.2. Organizational Dynamics

Teams have a unique opportunity to learn from the past attempts and adaptions of existing ERPs and consider complication pathways and triage pathways for post-discharge care. Emphasizing the learning that can come from recognizing the patterns that are more easily seen with standard practices, i.e., the ability to catch the signal from the noise. 

#### 2.2.1. Leadership

Leadership is critical for supporting probabilistic thinking and logical pattern recognition. Critical aspects of organizational culture [42] make a difference in how much a surgical practice can achieve. Collectively an active ‘just culture’ and a dynamic ‘continuous improvement’ culture are needed as individual team members may struggle with parts [57]. The just culture model allows for coaching and improvements while not honoring or coddling performance issues. The inter-professional, multidisciplinary team approach has become commonplace. The advantages are evident on a variety of fronts. The challenge remains that when everyone’s responsible, everyone is (duplicate work/waste at times) or no one is (breach of standards/potential safety risk). With teams, just as in team sports, everyone needs to know the game plan, yet not everyone does the same work. Clear roles and responsibilities need to be well articulated and then accountable as part of team-based care’s overall success [58,59].

#### 2.2.2. Diffusion

After initial implementation with colorectal surgeons in 2009 with minimally invasive teams, then expanded to the entire Division as a standard of practice for all patients undergoing colorectal surgery in 2011. Next, team members worked to diffuse to Gynecologic surgery [60], Breast [61], Urology [62,63,64], Endocrine, Hepatobiliary [65,66], Thoracic [67,68] and Vascular [69,70] within one campus of the institution, then continued to expand enterprise-wide knowledge within the same organization while also collaborating with teams at external organizations. The framework of spread was used, and results from network collaboratives shared [38]. Internal audits continue to drive quality improvement efforts for the targeted areas by specialty. Knowing critical factors [71] for predicting prolonged LOS and complications most relate to diet and fluid compliance. Additional opportunity exists across all teams for improved adoption of diet and fluid management principles.

### 2.3. Quality Improvement Methods

It cannot be discounted that optimal Quality Improvement methodologies are required to contextualize improvement efforts and assure full implementation, diffusion, and adoption has occurred and will continue to be sustained. Core QI methodologies strengthen the clinical programs and allow early recognition of issues. Like clinical care, the ‘pulse of the practice’ is the health and wellness of any patient care unit, Division, Department, or entire Health System ecosystem. Knowing the status (as a project management term) as the ‘health’ of the organization or program is how ongoing improvements, new research questions, and innovations can emerge readily.

The Design, Measure, Analyze, Improve, Control (DMAIC) model was used to strategize then implement the following tactics. The overall goal was to improve patients’ recovery plans that lead to improved patient hospital length of stay. The first was to review the current state of enhanced recovery compliance. It is well established that high compliance with enhanced recovery principles leads to better outcomes [43,64,72]. A report within the Electronic Health Record (EHR) was developed to implement a new health system to replace previous custom software dashboards and point of care tools related to ERP. The current compliance across the Colorectal Surgical Division remains >90% across the timeframe of the past two years, despite the transition to a new electronic health record. There is a slight variation between the Surgeons, yet no individual provider is below 80%. The various themes are consistent with previous emergency cases’ challenges and patients admitted through ED and medical service before going to surgery. When reviewing critical elements of the entire pathway, opportunities exist with intraoperative fluid [73,74,75]. When examining options postoperatively, reemphasizing diet compliance for all staff involved may help with additional compliance needs. Had there been a large discrepancy or actionable gap, our Practice Optimization team would have embarked on specific quality improvement tactics to improve. However, we recognized a more significant impact on two bookends of the current Practice. (1) opportunity to shift from Inpatient to Outpatient for specific procedures/patient population and (2) minimize issues related to an extended length of stay. The development of an outpatient enhanced recovery has been implemented, and the clinical outcomes of this are being collected in ongoing work and will be described in another paper. 

The refreshing of standardized plans for each day post-op has been outlined for the team and implemented daily multidisciplinary rounds. In parallel to the re-emphasis of standardized discharge criteria, daily accountability for discharge planning, and an active Practice Optimization and Acceleration project with the team, a baseline LOS from 2019 has been decreased by 1.4 days LOS to again move to early implementation LOS median of 4 days that we had reported before [37].

## 3. Complications

### 3.1. Post Operative Ileus (POI)

For POI, enhanced recovery principles are designed to prevent POI [4]. The literature on this is robust—minimize NPO preoperatively, no nasogastric tube (NGT) postoperatively, and regular diet provided within 4 h of surgery [4,76]. Earlier work had identified estimated blood loss and total opioid dose as independently associated with duration of POI in a pre-ERP era [77], which we assessed as technique related for EBL and actionable by opioid sparing methods covered with multi-modal pain control techniques. When ERP is implemented effectively with early feeding as critical for patient, it minimizes POI to low rates and in our practice reduces rates by a factor of 3 compared to not following this standard [78]. While medications, such as alvimopan, have been studied with promising results compared to traditional practice [79], no additional advantages have been proven in units with highly compliant enhanced recovery pathways. We do not include alvimopan in the active enhanced recovery pathway for colorectal surgery as it increases the cost of care, without providing added benefit in our patient population of highly compliant enhanced recovery. 

The other factor is to avoid fluid overload [36,46,73,75,80,81,82]. We continue to work with our surgical and anesthesiologist colleagues on achieving euvolemia and avoiding fluid overload to minimize POI and other complications.

### 3.2. Surgical Site Infection (SSI)

The increasing adoption of standardized ERP and growing rates of Minimally Invasive Surgery have reduced of the complication of SSI. Locally this has been supplemented by following high adherence to both ERP and a standardized SSI bundle [83]. Ongoing efforts to sustain high compliance with the bundle have continued. One controversy remains, which is the use of mechanical bowel prep with oral antibiotics before surgery. In the 2009 version of our enhanced recovery pathway, we instituted no bowel prep as the clinical standard [36]. During this high ERP compliance and standardized SSI bundle initiation, the SSI rates dropped and remained low [83]. With the Michigan Quality Improvement teams’ work, mechanical bowel prep with oral antibiotics showed great benefit [84]. While it’s unclear whether merely implementing a standard of practice with strict monitoring and key metrics being transparently shared had a Hawthorne effect or whether the evidence is translatable. No randomized control trial (RCT) in the era of enhanced recovery and minimally invasive surgery (MIS) has occurred, and the SSI rates are low, making a feasible RCT challenging to perform. Our institution’s power analysis with the following qualities and assumptions would require >17,000 patients for 90% power. Nationally, as MIS rates increase, the impact of bowel prep may not be as critical as once thought.

### 3.3. Acute Kidney Injury

There are various facets which if implemented may prevention acute kidney injury. Appropriately implemented criteria for the use of non-steroidal anti-inflammatory agents, such that patients with known renal dysfunction before surgery are not exposed can result in better outcomes. The topic continued to be a barrier to full implementation for specific team members and for others researching [85,86,87,88,89]. The key difference is pharmacists in our institution serve as the safety net for appropriate medication ordering as standardized order sets, and electronic decisions have been designed to help the initial ordering provider dose appropriately for these patients. By tracking the data as well as implementing the safe guard systems, we have not seen AKI as a barrier to implementation and rather a risk able to be mitigated [89].

Fluid overload preoperatively, in the OR, and postoperatively are to be avoided. One tactic, implemented through multiple Plan, Study, Do, Act (PSDA) cycles to improve fluid compliance, was that operationally, we changed to not starting IV fluids upon arrival to the facility day of surgery but instead waiting until the patient was in the OR. This operational change through orderset updates and preoperative nursing education focusing on allowing patients to drink fluids as per American Society of Regional Anesthesia and Pain Medicine (ASRA) standards rather than IV decreased the amount of fluid by approximately 1.5 L preoperatively based on internal audits. Within the OR, the fluids volumes have remained higher than our enhanced recovery pathway recommendations, and we see differences in outcomes when the goals are unable to be achieved [73]. Recent data suggest that total volume of fluid given rather than the rate in mL/Kg/hour is more important in our high-volume practice in terms of LOS and complication risk (paper in submission).

Postoperatively, the discontinuation of IV fluids at 0800 days after surgery has effectively reduced the amount of IV fluid exposure. However, the institution is still challenged by historical dogma to react to requests for more fluid from other team members. Low urine output, for example, is expected and not harmful in the colorectal patient population we serve, while fluid overload has been proven harmful. While there is still dogma for giving more fluid, our research shows that not only is a lower urinary output expected and acceptable in the early postoperative period [90], reactionary IV fluid and fluid overload were not beneficial [78]. In a study attempting to reverse the fluid overload situation with furosemide, there was no benefit, and the practice was ceased [91]. High ileostomy output has a known pathway for assuring the patient is eating adequately and the timing of increased production as a pattern in the postoperative setting to be expected and not overreacted to in the first 24–48 h postoperatively.

For each of the other surgical complications, we have developed and implemented programs for each topic. For example—VTE prophylaxis standards are embedded into the order sets and monitoring plans [92] and specific to colorectal surgery discharge plans [93,94]. A postoperative bleeding pathway was developed and implemented; a postoperative atrial fibrillation pathway was developed and implemented [95]. Considering the nation’s attention to the opioid crisis, we studied discharge prescriptions and the newly deemed complication of prolonged opioid use [59,60]. From the work, new discharge guidelines were established, and improved outpatient triage options were implemented. Each of these initiatives followed similar methods and quality improvement tactics for execution that were able to be disseminated [38].

### 3.4. Readmissions

To smooth implementation concerns, we measured readmission as a counterbalance in all cases. Others findings were informative [42,47,96,97]. In our reviews of readmission patterns, we recognized that the readmission risk continued to rise with each added hospital day, i.e., the longer length of stays correlated with high readmissions We studied the disease state patterns and complications for each as a guide for learners to see those patterns sooner. The probability of predicting a patient’s trajectory became important as we attempt to keep patients informed about reasonable expectations [98].

The key themes are these: (a) A longer length of stay was correlated with a higher rate of readmission; (b) Once the patients meet our standard discharge criteria, there is no further advantage of inpatient/hospital care and (c) logistical barriers are known where at times, a patient needing a skilled nursing facility may not get placement for several days after meeting discharge criteria clinically with the surgical team. This remains a challenge both within and outside of our health care system.

## 4. Sustainability

As described in ERP implementation work [1,64] an organizational framework is needed to sustain the gains [44,48]. Ongoing work to display the required actionable information in the clinical workflows within the electronic health record. Overall compliance is known with reports, and the length of stay targets have been presented to the team leads with dashboard functionality. The themes for electronic tools are consistent with design principles for putting the information needed for decision-making in the decision-maker’s hands when the decision needs to be made [99]. Tactically, this means standardized order sets for pre- and post-operative care, real-time patient information collated to simple list views for rounding and monitoring teams, and leadership reports for high-level summaries.

## 5. Future

Our next phase of research and implementation will expand on outpatient care opportunities for segmental colectomy patients. Moreover, advancing minimally invasive techniques, improving fluid management, and continuing to work on complication pathways for the chance to provide high-value care to our patients and decrease morbidity.

## 6. Conclusions

Enhanced recovery improves care for patients and allows optimal standardization for institutions and care teams for optimal systematic approaches to excellence in patient care. We share the framework and experiences so that others may partner with their teams or ours to achieve more. After over a decade, compliance remains high. Ongoing innovations, essential quality improvement methods, and continued opportunities remain challenging work to pursue.

## Data Availability

No new data were created or analyzed in this study. Data sharing is not applicable to this article.

## References

[1] Bonnet F., Rousset J. (2012). Regional anaesthesia in fast track surgery and rehabilitation. Reg. Anesth. Pain Med..

[2] Tsikitis V.L., Holubar S.D., Dozois E.J., Cima R.R., Pemberton J.H., Larson D.W. (2010). Advantages of fast-track recovery after laparoscopic right hemicolectomy for colon cancer. Surg. Endosc..

[3] Varadhan K.K., Lobo D.N., Ljungqvist O. (2010). Enhanced recovery after surgery: The future of improving surgical care. Crit. Care Clin..

[4] Kehlet H. (1997). Multimodal approach to control postoperative pathophysiology and rehabilitation. Br. J. Anaesth..

[5] Ljungqvist O., Scott M., Fearon K.C. (2017). Enhanced Recovery After Surgery: A Review. JAMA Surg..

[6] Brindle M., Nelson G., Lobo D.N., Ljungqvist O., Gustafsson U.O. (2020). Recommendations from the ERAS^®^ Society for standards for the development of enhanced recovery after surgery guidelines. BJS Open.

[7] Ljungqvist O., Young-Fadok T., Demartines N. (2017). The History of Enhanced Recovery After Surgery and the ERAS Society. J. Laparoendosc. Adv. Surg. Tech. A.

[8] Elias K.M., Stone A.B., McGinigle K., Tankou J., Scott M.J., Fawcett W.J., Demartines N., Lobo D.N., Ljungqvist O., Urman R.D. (2019). The Reporting on ERAS Compliance, Outcomes, and Elements Research (RECOvER) Checklist: A Joint Statement by the ERAS^®^ and ERAS^®^ USA Societies. World J. Surg..

[9] Ljungqvist O., Thanh N.X., Nelson G. (2017). ERAS-Value based surgery. J. Surg. Oncol..

[10] Tanious M.K., Ljungqvist O., Urman R.D. (2017). Enhanced Recovery After Surgery: History, Evolution, Guidelines, and Future Directions. Int. Anesthesiol. Clin..

[11] Currie A., Soop M., Demartines N., Fearon K., Kennedy R., Ljungqvist O. (2019). Enhanced Recovery After Surgery Interactive Audit System: 10 Years’ Experience with an International Web-Based Clinical and Research Perioperative Care Database. Clin. Colon Rectal Surg..

[12] Loughlin S.M., Alvarez A., Falcão L.F.D.R., Ljungqvist O. (2020). The History of ERAS (Enhanced Recovery After Surgery) Society and its development in Latin America. Rev. Col. Bras. Cir..

[13] Pisarska M., Torbicz G., Gajewska N., Rubinkiewicz M., Wierdak M., Major P., Budzyński A., Ljungqvist O., Pędziwiatr M. (2019). Compliance with the ERAS Protocol and 3-Year Survival After Laparoscopic Surgery for Non-metastatic Colorectal Cancer. World J. Surg..

[14] Nelson G., Bakkum-Gamez J., Kalogera E., Glaser G., Altman A., Meyer L.A., Taylor J.S., Iniesta M., Lasala J., Mena G. (2019). Guidelines for perioperative care in gynecologic/oncology: Enhanced Recovery after Surgery (ERAS) Society recommendations–2019 update. Int. J. Gynecol. Cancer.

[15] Caughey A.B., Wood S.L., Macones G.A., Wrench I.J., Huang J., Norman M., Pettersson K., Fawcett W.J., Shalabi M.M., Metcalfe A. (2018). Guidelines for intraoperative care in cesarean delivery: Enhanced Recovery After Surgery Society Recommendations (Part 2). Am. J. Obstet. Gynecol..

[16] Engelman D.T., Ben Ali W., Williams J.B., Perrault L.P., Reddy V.S., Arora R.C., Roselli E.E., Khoynezhad A., Gerdisch M., Levy J.H. (2019). Guidelines for Perioperative Care in Cardiac Surgery: Enhanced Recovery after Surgery Society Recommendations. JAMA Surg..

[17] Gibb A.C.N., Crosby M.A., McDiarmid C., Urban D., Lam J.Y.K., Wales P.W., Brockel M., Raval M., Offringa M., Skarsgard E.D. (2018). Creation of an Enhanced Recovery after Surgery (ERAS) Guideline for neonatal intestinal surgery patients: A knowledge synthesis and consensus generation approach and protocol study. BMJ Open.

[18] Nygren J., Thacker J., Carli F., Fearon K.C.H., Norderval S., Lobo D.N., Ljungqvist O., Soop M., Ramirez J. (2012). Guidelines for perioperative care in elective rectal/pelvic surgery: Enhanced Recovery After Surgery (ERAS^®^) Society recommendations. Clin. Nutr..

[19] Gustafsson U.O., Scott M.J., Schwenk W., Demartines N., Roulin D., Francis N., McNaught C.E., MacFie J., Liberman A.S., Soop M. (2012). Guidelines for perioperative care in elective colonic surgery: Enhanced Recovery After Surgery (ERAS^®^) Society recommendations. Clin. Nutr..

[20] Miller T.E., Thacker J.K., White W.D., Mantyh C., Migaly J., Jin J., Roche A.M., Eisenstein E.L., Edwards R., Anstrom K.J. (2014). Reduced length of hospital stay in colorectal surgery after implementation of an enhanced recovery protocol. Anesth. Analg..

[21] Fischer C., Wick E. (2020). An AHRQ national quality improvement project for implementation of enhanced recovery after surgery. Semin. Colon Rectal Surg..

[22] Brethauer S.A., Grieco A., Fraker T., Evans-Labok K., Smith A., McEvoy M.D., Saber A.A., Morton J.M., Petrick A. (2019). Employing Enhanced Recovery Goals in Bariatric Surgery (ENERGY): A national quality improvement project using the Metabolic and Bariatric Surgery Accreditation and Quality Improvement Program. Surg. Obes. Relat. Dis..

[23] Heathcote S.S., Duggan K., Rosbrugh J., Hill B., Shaker R., Hope W.W., Fillion M.M. (2019). Enhanced Recovery after Surgery (ERAS) Protocols Expanded over Multiple Service Lines Improves Patient Care and Hospital Cost. Am Surg..

[24] Pachella L.A., Mehran R.J., Curtin K., Schneider S.M. (2019). Preoperative Carbohydrate Loading in Patients Undergoing Thoracic Surgery: A Quality-Improvement Project. J. PeriAnesthesia Nurs..

[25] Liu J.Y., Wick E.C. (2018). Enhanced Recovery After Surgery and Effects on Quality Metrics. Surg. Clin. North Am..

[26] Jakobsen D.H., Kehle H. (2020). A simple method to secure data-driven improvement of perioperative care. Br. J. Nurs..

[27] Beverly A., Kaye A.D., Ljungqvist O., Urman R.D. (2017). Essential Elements of Multimodal Analgesia in Enhanced Recovery After Surgery (ERAS) Guidelines. Anesthesiol. Clin..

[28] Wick E.C., Grant M.C., Wu C.L. (2017). Postoperative Multimodal Analgesia Pain Management With Nonopioid Analgesics and Techniques: A Review. JAMA Surg..

[29] Simpson J.C., Bao X., Agarwala A. (2019). Pain Management in Enhanced Recovery after Surgery (ERAS) Protocols. Clin. Colon Rectal Surg..

[30] Mitra S., Carlyle D., Kodumudi G., Kodumudi V., Vadivelu N. (2018). New Advances in Acute Postoperative Pain Management. Curr. Pain Headache Rep..

[31] Weimann A., Braga M., Carli F., Higashiguchi T., Hübner M., Klek S., Laviano A., Ljungqvist O., Lobo D.N., Martindale R. (2017). ESPEN guideline: Clinical nutrition in surgery. Clin. Nutr..

[32] Gianotti L., Sandini M., Romagnoli S., Carli F., Ljungqvist O. (2020). Enhanced recovery programs in gastrointestinal surgery: Actions to promote optimal perioperative nutritional and metabolic care. Clin. Nutr..

[33] Mc Loughlin S., Terrasa S.A., Ljungqvist O., Sanchez G., Garcia Fornari G., Alvarez A.O. (2019). Nausea and vomiting in a colorectal ERAS program: Impact on nutritional recovery and the length of hospital stay. Clin. Nutr. ESPEN.

[34] Aahlin E.K., von Meyenfeldt M., Dejong C.H., Ljungqvist O., Fearon K.C., Lobo D.N., Demartines N., Revhaug A., Wigmore S.J., Lassen K. (2014). Functional recovery is considered the most important target: A survey of dedicated professionals. Perioper. Med..

[35] Maessen J., Dejong C.H.C., Hausel J., Nygren J., Lassen K., Andersen J., Kessels A.G.H., Revhaug A., Kehlet H., Ljungqvist O. (2007). A protocol is not enough to implement an enhanced recovery programme for colorectal resection. Br. J. Surg..

[36] Lovely J.K., Maxson P.M., Jacob A.K., Cima R.R., Horlocker T.T., Hebl J.R., Harmsen W.S., Huebner M., Larson D.W. (2012). Case-matched series of enhanced versus standard recovery pathway in minimally invasive colorectal surgery. Br. J. Surg..

[37] Larson D.W., Lovely J.K., Cima R.R., Dozois E.J., Chua H., Wolff B.G., Pemberton J.H., Devine R.R., Huebner M. (2014). Outcomes after implementation of a multimodal standard care pathway for laparoscopic colorectal surgery. Br. J. Surg..

[38] Larson D.W., Lovely J.K., Welsh J., Annaberdyev S., Corning Coffey C., Murray B., Rose D., Prabhakar L., Torgenson M., Dankbar E. (2018). A Collaborative for Implementation of an Evidence-Based Clinical Pathway for Enhanced Recovery in Colon and Rectal Surgery in an Affiliated Network of Healthcare Organizations. Jt. Comm. J. Qual. Patient Saf..

[39] Quiram B.J., Crippa J., Grass F., Lovely J.K., Behm K.T., Colibaseanu D.T., Merchea A., Kelley S.R., Harmsen W.S., Larson D.W. (2019). Impact of enhanced recovery on oncological outcomes following minimally invasive surgery for rectal cancer. Br. J. Surg..

[40] Khreiss W., Huebner M., Cima R.R., Dozois E.R., Chua H.K., Pemberton J.H., Harmsen W.S., Larson D.W. (2014). Improving conventional recovery with enhanced recovery in minimally invasive surgery for rectal cancer. Dis. Colon Rectum.

[41] Elhassan A., Ahmed A., Awad H., Humeidan M., Nguyen V., Cornett E.M., Urman R.D., Kaye A.D. (2018). The Evolution of Surgical Enhanced Recovery Pathways: A Review. Curr. Pain Headache Rep..

[42] Slakey D.P., Silver D.S., Chazin S.M., Katoozian P.Y., Sikora K.S., Ruther M.M. (2020). Making enhanced recovery the norm not the exception. Am. J. Surg..

[43] Williamsson C., Karlsson T., Westrin M., Ansari D., Andersson R., Tingstedt B. (2019). Sustainability of an Enhanced Recovery Program for Pancreaticoduodenectomy with Pancreaticogastrostomy. Scand. J. Surg..

[44] Wolfe D., Knighton A.J., Brunisholz K.D., Belnap T., Allen T.L., Srivastava R. (2019). Sustaining Implementation Gains. Qual. Manag. Health Care.

[45] Li N., Laux C.M., Antony J. (2019). How to use lean Six Sigma methodology to improve service process in higher education: A case study. Int. J. Lean Six Sigma.

[46] Joliat G.R., Ljungqvist O., Wasylak T., Peters O., Demartines N. (2018). Beyond surgery: Clinical and economic impact of Enhanced Recovery after Surgery programs. BMC Health Serv. Res..

[47] Fabrizio A.C., Grant M.C., Siddiqui Z., Alimi Y., Gearhart S.L., Wu C., Efron J.E., Wick E.C. (2017). Is enhanced recovery enough for reducing 30-d readmissions after surgery?. J. Surg. Res..

[48] Stone A.B., Yuan C.T., Rosen M.A., Grant M.C., Benishek L.E., Hanahan E., Lubomski L.H., Ko C., Wick E.C. (2018). Barriers to and facilitators of implementing enhanced recovery pathways using an implementation framework: A systematic review. JAMA Surg..

[49] Li D., Jensen C.C. (2019). Patient Satisfaction and Quality of Life with Enhanced Recovery Protocols. Clin. Colon Rectal Surg..

[50] Currie A., Burch J., Jenkins J.T., Faiz O., Kennedy R.H., Ljungqvist O., Demartines N., Hjern F., Norderval S., Lassen K. (2015). The impact of enhanced recovery protocol compliance on elective colorectal cancer resection: Results from an international registry. Ann. Surg..

[51] Gramlich L.M., Sheppard C.E., Wasylak T., Gilmour L.E., Ljungqvist O., Basualdo-Hammond C., Nelson G. (2017). Implementation of Enhanced Recovery After Surgery: A strategy to transform surgical care across a health system. Implement. Sci..

[52] Grass F., Crippa J., Mathis K.L., Kelley S.R., Larson D.W. (2019). Feasibility and safety of robotic resection of complicated diverticular disease. Surg. Endosc..

[53] Lemini R., Spaulding A.C., Naessens J.M., Li Z., Merchea A., Crook J.E., Larson D.W., Colibaseanu D.T. (2018). ERAS protocol validation in a propensity-matched cohort of patients undergoing colorectal surgery. Int. J. Colorectal Dis..

[54] Huebner M., Hübner M., Cima R.R., Larson D.W. (2014). Timing of complications and length of stay after rectal cancer surgery. J. Am. Coll. Surg..

[55] Lovely J.K., Hyland S.J., Smith A.N., Nelson G., Ljungqvist O., Parrish R.H. (2019). Clinical pharmacist perspectives for optimizing pharmacotherapy within Enhanced Recovery After Surgery (ERAS^®^) programs. Int. J. Surg..

[56] Patel G.P., Hyland S.J., Birrer K.L., Wolfe R.C., Lovely J.K., Smith A.N., Dixon R.L., Johnson E.G., Gaviola M.L., Giancarelli A. (2020). Perioperative clinical pharmacy practice: Responsibilities and scope within the surgical care continuum. J. Am. Coll. Clin. Pharm..

[57] Swensen S.J., Dilling J.A., Milliner D.S., Zimmerman R.S., Maples W.J., Lindsay M.E., Bartley G.B. (2009). Quality: The Mayo Clinic approach. Am. J. Med. Qual..

[58] Burton R.M., Obel B. (2018). The science of organizational design: Fit between structure and coordination. J. Organ. Des..

[59] Timmel J., Kent P.S., Holzmueller C.G., Paine L., Schulick R.D., Pronovost P.J. (2010). Impact of the Comprehensive Unit-based Safety Program (CUSP) on safety culture in a surgical inpatient unit. Jt. Comm. J. Qual. Patient Saf..

[60] Kalogera E., Bakkum-Gamez J.N., Jankowski C.J., Trabuco E., Lovely J.K., Dhanorker S., Grubbs P.L., Weaver A.L., Haas L.R., Borah B.J. (2013). Enhanced recovery in gynecologic surgery. Obstet. Gynecol..

[61] Batdorf N.J., Lemaine V., Lovely J.K., Ballman K.V., Goede W.J., Martinez-Jorge J., Booth-Kowalczyk A.L., Grubbs P.L., Bungum L.D., Saint-Cyr M. (2015). Enhanced recovery after surgery in microvascular breast reconstruction. J. Plast. Reconstr. Aesthetic Surg..

[62] Williams S.B., Cumberbatch M.G.K., Kamat A.M., Jubber I., Kerr P.S., McGrath J.S., Djaladat H., Collins J.W., Packiam V.T., Steinberg G.D. (2020). Reporting Radical Cystectomy Outcomes Following Implementation of Enhanced Recovery After Surgery Protocols: A Systematic Review and Individual Patient Data Meta-analysis. Eur. Urol..

[63] Cerantola Y., Valerio M., Persson B., Jichlinski P., Ljungqvist O., Hubner M., Kassouf W., Muller S., Baldini G., Carli F. (2013). Guidelines for perioperative care after radical cystectomy for bladder cancer: Enhanced recovery after surgery (ERAS^®^) society recommendations. Clin. Nutr..

[64] Carter-Brooks C.M., Du A.L., Ruppert K.M., Romanova A.L., Zyczynski H.M. (2018). Implementation of a urogynecology-specific enhanced recovery after surgery (ERAS) pathway. Am. J. Obstet. Gynecol..

[65] Agarwal V., Divatia J.V. (2019). Enhanced recovery after surgery in liver resection: Current concepts and controversies. Korean J. Anesthesiol..

[66] Melloul E., Hübner M., Scott M., Snowden C., Prentis J., Dejong C.H.C., Garden O.J., Farges O., Kokudo N., Vauthey J.N. (2016). Guidelines for Perioperative Care for Liver Surgery: Enhanced Recovery After Surgery (ERAS) Society Recommendations. World J. Surg..

[67] Low D.E., Allum W., De Manzoni G., Ferri L., Immanuel A., Kuppusamy M.K., Law S., Lindblad M., Maynard N., Neal J. (2019). Guidelines for Perioperative Care in Esophagectomy: Enhanced Recovery After Surgery (ERAS^®^) Society Recommendations. World J. Surg..

[68] Batchelor T.J.P., Rasburn N.J., Abdelnour-Berchtold E., Brunelli A., Cerfolio R.J., Gonzalez M., Ljungqvist O., Petersen R.H., Popescu W.M., Slinger P.D. (2019). Guidelines for enhanced recovery after lung surgery: Recommendations of the Enhanced Recovery after Surgery (ERAS^®^) Society and the European Society of Thoracic Surgeons (ESTS). Eur. J. Cardio-Thorac. Surg..

[69] Brustia P., Renghi A., Aronici M., Gramaglia L., Porta C., Musiani A., Martelli M., Casella F., De Simeis M.L., Coppi G. (2015). Fast-track in abdominal aortic surgery: Experience in over 1000 patients. Ann. Vasc. Surg..

[70] Witcher A., Axley J., Novak Z., Laygo-Prickett M., Guthrie M., Xhaja A., Chu D.I., Brokus S.D., Spangler E.L., Passman M.A. (2021). Implementation of an enhanced recovery program for lower extremity bypass. J. Vasc. Surg..

[71] Huebner M., Larson D.W., Cima R.R., Habermann E. (2013). 785 Impact of Key Factors of Enhanced Recovery Pathway and Preexisting Comorbidities on Complications and Length of Stay Following Colorectal Surgery. Gastroenterology.

[72] Paton F., Chambers D., Wilson P., Eastwood A., Craig D., Fox D., Jayne D., McGinnes E. (2014). Effectiveness and implementation of enhanced recovery after surgery programmes: A rapid evidence synthesis. BMJ Open.

[73] Grass F., Lovely J.K., Crippa J., Hübner M., Mathis K.L., Larson D.W. (2020). Potential association between perioperative fluid management and occurrence of postoperative ileus. Dis. Colon Rectum.

[74] Zhu A.C.C., Agarwala A., Bao X. (2019). Perioperative Fluid Management in the Enhanced Recovery after Surgery (ERAS) Pathway. Clin. Colon Rectal Surg..

[75] Grass F., Hübner M., Mathis K.L., Hahnloser D., Dozois E.J., Kelley S.R., Demartines N., Larson D.W. (2020). Challenges to accomplish stringent fluid management standards 7 years after enhanced recovery after surgery implementation—The surgeon’s perspective. Surgery (United States).

[76] Bisch S., Nelson G., Altman A. (2019). Impact of nutrition on enhanced recovery after surgery (ERAS) in gynecologic oncology. Nutrients.

[77] Artinyan A., Nunoo-Mensah J.W., Balasubramaniam S., Gauderman J., Essani R., Gonzalez-Ruiz C., Kaiser A.M., Beart R.W. (2008). Prolonged postoperative ileus - Definition, risk factors, and predictors after surgery. World J. Surg..

[78] Grass F., Hübner M., Lovely J.K., Crippa J., Mathis K.L., Larson D.W. (2018). Ordering a normal diet at the end of surgery—justified or overhasty?. Nutrients.

[79] Kelley S.R., Wolff B.G., Lovely J.K., Larson D.W. (2013). Fast-track pathway for minimally invasive colorectal surgery with and without alvimopan (entereg)^TM^: Which is more cost-effective?. Am. Surg..

[80] Chen F., Rasouli M.R., Ellis A.R., Ohnuma T., Bartz R.R., Krishnamoorthy V., Haines K.L., Raghunathan K. (2020). Associations Between Perioperative Crystalloid Volume and Adverse Outcomes in Five Surgical Populations. J. Surg. Res..

[81] Evans T., Koek S., Ballal M. (2018). Perioperative restrictive intravenous fluid therapy in eras in pancreaticoduodenectomy. HPB.

[82] Guan Z., Gao Y., Qiao Q., Wang Q., Liu J. (2021). Effects of intraoperative goal-directed fluid therapy and restrictive fluid therapy combined with enhanced recovery after surgery protocol on complications after thoracoscopic lobectomy in high-risk patients: Study protocol for a prospective randomized controlled trial. Trials.

[83] Cima R., Dankbar E., Lovely J., Pendlimari R., Aronhalt K., Nehring S., Hyke R., Tyndale D., Rogers J., Quast L. (2013). Colorectal surgery surgical site infection reduction program: A national surgical quality improvement program-driven multidisciplinary single-institution experience. J. Am. Coll. Surg..

[84] Waits S.A., Fritze D., Banerjee M., Zhang W., Kubus J., Englesbe M.J., Campbell D.A., Hendren S. (2014). Developing an argument for bundled interventions to reduce surgical site infection in colorectal surgery. Surgery (United States).

[85] Hollis R.H., Kennedy G.D. (2020). Postoperative Complications After Colorectal Surgery: Where Are We in the Era of Enhanced Recovery?. Curr. Gastroenterol. Rep..

[86] Wiener J.G.D., Goss L., Wahl T.S., Terry M.A., Burge K.G., Chu D.I., Richman J.S., Cannon J., Kennedy G.D., Morris M.S. (2020). The association of enhanced recovery pathway and acute kidney injury in patients undergoing colorectal surgery. Dis. Colon Rectum.

[87] Hassinger T.E., Turrentine F.E., Thiele R.H., Sarosiek B.M., McMurry T.L., Friel C.M., Hedrick T.L. (2018). Acute kidney injury in the age of enhanced recovery protocols. Dis. Colon Rectum.

[88] Hanna P.T., Peterson M., Albersheim J., Drawz P., Zabell J., Konety B., Weight C. (2020). Acute Kidney Injury following Enhanced Recovery after Surgery in Patients Undergoing Radical Cystectomy. J. Urol..

[89] Grass F., Lovely J.K., Crippa J., Mathis K.L., Hübner M., Larson D.W. (2019). Early Acute Kidney Injury Within an Established Enhanced Recovery Pathway: Uncommon and Transitory. World J. Surg..

[90] Hübner M., Lovely J.K., Huebner M., Slettedahl S.W., Jacob A.K., Larson D.W. (2013). Intrathecal analgesia and restrictive perioperative fluid management within enhanced recovery pathway: Hemodynamic implications. J. Am. Coll. Surg..

[91] Danelich I.M., Bergquist J.R., Bergquist W.J., Osborn J.L., Wright S.S., Tefft B.J., Sturm A.W., Langworthy D.R., Mandrekar J., Devine R.M. (2018). Early diuresis after colon and rectal surgery does not reduce length of hospital stay: Results of a randomized trial. Dis. Colon Rectum.

[92] Morgenthaler T.I., Lovely J.K., Cima R.R., Berardinelli C.F., Fedraw L.A., Wallerich T.J., Hinrichs D.J., Varkey P. (2012). Using a framework for spread of best practices to implement successful venous thromboembolism prophylaxis throughout a large hospital system. Am. J. Med. Qual..

[93] McKenna N.P., Shariq O.A., Bews K.A., Mathis K.L., Lightner A.L. (2018). Venous thromboembolism in inflammatory bowel disease: Is it the disease, the operation, or both?. Dis. Colon Rectum.

[94] McKenna N.P., Bews K.A., Behm K.T., Mathis K.L., Lightner A.L., Habermann E.B. (2020). Do patients with inflammatory bowel disease have a higher postoperative risk of venous thromboembolism or do they undergo more high-risk operations?. Ann. Surg..

[95] Danelich I.M., Lose J.M., Wright S.S., Asirvatham S.J., Ballinger B.A., Larson D.W., Lovely J.K. (2014). Practical management of postoperative atrial fibrillation after noncardiac surgery. J. Am. Coll. Surg..

[96] Ostermann S., Morel P., Chalé J.J., Bucher P., Konrad B., Meier R.P.H., Ris F., Schiffer E.R.C. (2019). Randomized Controlled Trial of Enhanced Recovery Program Dedicated to Elderly Patients after Colorectal Surgery. Dis. Colon Rectum.

[97] Chin A.B., Galante D.J., Hobson D.B., Efron J.E., Gearhart S.L., Safar B., Fang S.H., Wu C., Wick E.C. (2015). Readmission after Enhanced Recovery in Colorectal Patients. J. Am. Coll. Surg..

[98] Grass F., Crippa J., Lovely J.K., Ansell J., Behm K.T., Achilli P., Hübner M., Kelley S.R., Mathis K.L., Dozois E.J. (2020). Readmissions within 48 Hours of Discharge: Reasons, Risk Factors, and Potential Improvements. Dis. Colon Rectum.

[99] Michard F., Gan T.J., Kehlet H. (2017). Digital innovations and emerging technologies for enhanced recovery programmes. Br. J. Anaesth..

